# A Network Biology Approach to Understanding the Tissue-Specific Roles of Non-Coding RNAs in Arthritis

**DOI:** 10.3389/fendo.2021.744747

**Published:** 2021-11-03

**Authors:** Shabana Amanda Ali, Chiara Pastrello, Navdeep Kaur, Mandy J. Peffers, Michelle J. Ormseth, Igor Jurisica

**Affiliations:** ^1^ Bone and Joint Center, Department of Orthopaedic Surgery, Henry Ford Health System, Detroit, MI, United States; ^2^ Center for Molecular Medicine and Genetics, Wayne State University, Detroit, MI, United States; ^3^ Osteoarthritis Research Program, Division of Orthopaedics, Schroeder Arthritis Institute, University Health Network, Toronto, ON, Canada; ^4^ Data Science Discovery Centre for Chronic Diseases, Krembil Research Institute, University Health Network, Toronto, ON, Canada; ^5^ Department of Musculoskeletal Biology, Institute of Life Course and Medical Sciences, University of Liverpool, Liverpool, United Kingdom; ^6^ Department of Research and Development, Veterans Affairs Medical Center, Nashville, TN, United States; ^7^ Department of Medical Biophysics, University of Toronto, Toronto, ON, Canada; ^8^ Department of Computer Science, University of Toronto, Toronto, ON, Canada; ^9^ Institute of Neuroimmunology, Slovak Academy of Sciences, Bratislava, Slovakia

**Keywords:** epigenetics, microRNA, long non-coding RNA, circular RNA, cartilage, synovium, integrative computational biology, network analysis and visualization

## Abstract

Discovery of non-coding RNAs continues to provide new insights into some of the key molecular drivers of musculoskeletal diseases. Among these, microRNAs have received widespread attention for their roles in osteoarthritis and rheumatoid arthritis. With evidence to suggest that long non-coding RNAs and circular RNAs function as competing endogenous RNAs to sponge microRNAs, the net effect on gene expression in specific disease contexts can be elusive. Studies to date have focused on elucidating individual long non-coding-microRNA-gene target axes and circular RNA-microRNA-gene target axes, with a paucity of data integrating experimentally validated effects of non-coding RNAs. To address this gap, we curated recent studies reporting non-coding RNA axes in chondrocytes from human osteoarthritis and in fibroblast-like synoviocytes from human rheumatoid arthritis. Using an integrative computational biology approach, we then combined the findings into cell- and disease-specific networks for in-depth interpretation. We highlight some challenges to data integration, including non-existent naming conventions and out-of-date databases for non-coding RNAs, and some successes exemplified by the International Molecular Exchange Consortium for protein interactions. In this perspective article, we suggest that data integration is a useful *in silico* approach for creating non-coding RNA networks in arthritis and prioritizing interactions for further *in vitro* and *in vivo* experimentation in translational research.

## Introduction

Noncoding RNAs are key regulators of gene expression in the musculoskeletal system. These RNA molecules are transcribed from DNA but not translated into protein (1). Among the many types of non-coding RNAs, the most characterized to date are microRNAs (miRNAs), long non-coding RNAs (lncRNAs), and circular RNAs (circRNAs). Through seed-sequence binding, miRNAs target specific genes and prevent their translation, effectively inhibiting expression. Because lncRNAs and circRNAs are both capable of ‘sponging’ miRNAs, or competitively binding to miRNAs, they are considered competing endogenous RNAs (ceRNAs) (2). LncRNAs function by binding to miRNAs through miRNA Response Elements (MRE) present at their 3’ ends (3). Therefore, lncRNAs act as molecular decoys, sequestering miRNAs and preventing their interaction with gene targets. As with circRNAs, lncRNAs regulate diverse biological processes through their crosstalk with miRNAs (4). This ceRNA activity is one of many factors contributing to the net effect of miRNAs in a specific biological context.

There are multiple factors governing miRNA expression, from the level of the cell to the organism. MiRNA biogenesis involves several post-transcriptional processing steps and is under both temporal and spatial control that can introduce variability (5). Once processed, mature miRNAs may decay, be degraded, be exported, or be ‘sponged’ as described above. As a result, the miRNAs present in a given cell can vary according to these regulatory factors, but also multiple biological factors, including age, sex, and body mass index (6). It is therefore not surprising that miRNA profiles are dysregulated in several disease contexts and contribute to disease onset and progression. For highly prevalent musculoskeletal diseases like osteoarthritis (OA) and rheumatoid arthritis (RA), multiple tissues in the joint compartment can show pathology that is driven by tissue-specific miRNA patterns. This multi-level regulation of miRNA expression presents a challenge when attempting to elucidate the function of a particular miRNA in disease.

MiRNAs play both protective and destructive roles in the musculoskeletal system (7). Since a miRNA can have hundreds of gene targets, its specific effect can be obscure. Studies to date have tended to focus on elucidating a single axis involving one or two non-coding RNAs, often a lncRNA or circRNA, and its effects on a miRNA and one or two downstream gene targets. While these studies are necessary to demonstrate direct interactions, they may be missing the broader biological context. In fact, the same miRNAs are often reported as having important functions through completely different pathways that involve unique upstream regulators and downstream effectors. Taking miR-140 as an example, independent studies have investigated its effects on at least 17 different gene targets in various cell types within the musculoskeletal system (8). Though it is now generally accepted that miR-140 plays a beneficial role in OA, promoting cartilage anabolism and inhibiting catabolism, this information was acquired over a decade of research from multiple groups, and remains under investigation due to its tissue- and disease-specific effects. For example in RA, miR-140 has been reported to suppress synovial inflammation (9), again suggesting a beneficial role, but in a different tissue and through a different mechanism than for OA, thereby precluding direct comparison of this miRNA across diseases.

To improve our understanding of the biological and clinical context of non-coding RNA regulation in arthritis, there is an outstanding need for methods to identify the interactions among non-coding RNAs and their gene targets in specific cell types. Computational biology approaches to predict ceRNA activity among non-coding RNAs, as well as gene targets and pathways, is a useful *in silico* strategy that can be tailored to cells, diseases, and other factors of interest. To illustrate this, we present two examples that integrate the recent literature on non-coding RNAs in OA and RA, focusing on chondrocytes and fibroblast-like synoviocytes (FLS), respectively. These examples are proof-of-concept of the relationships (or biological networks) that can be constructed using non-coding RNA axes that have been experimentally validated. In building these networks, we encountered key challenges, including inconsistency in annotation of non-coding RNAs. These challenges are discussed along with our perspectives on future directions needed to advance the non-coding RNA field.

## Methods

To curate relevant literature reporting non-coding RNA axes in OA chondrocytes and RA FLS, we searched PubMed using combinations of key words including osteoarthritis, rheumatoid arthritis, microRNA, long non-coding RNA, and circular RNA. Articles were filtered for those with an experimentally validated lncRNA-miRNA-mRNA or circRNA-miRNA-mRNA axis in chondrocytes from OA subjects or FLS from RA subjects. We included 36 studies for OA and 10 studies for RA published between October 2019 and December 2020. The lncRNA/circRNA, miRNA, and mRNA targets were extracted from individual papers. Common groups of genes were fed into a network prepared in NAViGaTOR v3.0.14 (10), connecting them using physical protein-protein interactions to visualize potential signaling relationships. The network figure was finalized with legend from an exported SVG file in Adobe Illustrator v25.3.1. Network source files are available upon request. Physical protein interactions among gene targets were obtained from Integrated Interactions Database (IID) v2021-05, using the entire set of IID interactions (11). Proteins were annotated with Gene Ontology (GO) biological processes using NAViGaTOR plugin to UniProt. The GO biological processes are represented in the figures by node color and are listed in the figure legends.

## Non-coding RNA Network in OA Chondrocytes

**Figure 1:**
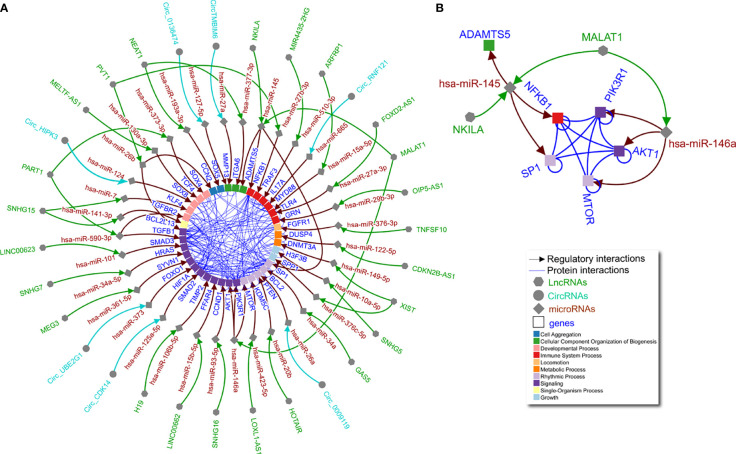
Non-coding RNA network in OA chondrocytes. **(A)** Literature curated lncRNA-miRNA-gene and circRNA-miRNA-gene axes (green, turquoise, and brown arrows) complemented by physical protein interactions (blue nondirectional lines connecting gene products) showing an interconnected regulatory network of non-coding RNAs in human OA chondrocytes. Node shape represents different molecule types, while gene target color signifies Gene Ontology biological process (as indicated in the legend). **(B)** The largest connected subgraph from panel A considering only literature curated non-coding, directed interactions and including undirected protein interaction connections among the pertinent gene targets.

OA is a degenerative disease of the joints, causing pain and disability in 7% of the global population (12). Pathology is most commonly observed in the cartilage, synovium, and bone, but OA research has by far focused on cartilage, including exploration of the role of non-coding RNAs (7). Dysregulation of miRNAs, lncRNAs, and circRNAs has been reported in OA, with one molecule having effects on multiple downstream molecules (e.g., mRNAs), including other non-coding RNAs (2). These non-coding RNA-based regulatory networks represent a molecular mechanism underlying OA that remains poorly understood. The majority of studies we identified explored a single non-coding RNA axis in OA chondrocytes, with several studies reporting on the same non-coding RNAs, but in different axes, making it difficult to ascertain the net effect of a particular molecule. To gain a better understanding of the networks in which non-coding RNAs function in OA, we used a computational biology approach to integrate these individual axes (**Figure 1A**; **Supplementary Table 1**).

As shown in **Figure 1A**, individual miRNAs, lncRNAs, and circRNAs in OA chondrocytes have multiple and interconnected effects in regulating downstream molecules. For example, a single lncRNA can affect multiple miRNAs as shown by lncRNA MALAT1 *via* miR-146a and miR-145 (**Figure 1B**). In lipopolysaccharide-treated chondrocytes, MALAT1/miR-146a modulates extracellular matrix catabolism, inflammation, and apoptosis through PI3K/Akt/mTOR, impacting OA progression (13). In addition, in interleukin (IL)-1β-treated chondrocytes, MALAT1/miR-145 increases extracellular matrix degradation by targeting a disintegrin and metalloproteinase with thrombospondin motifs 5 (ADAMTS5) (14). Furthermore, lncRNA NKILA may compete for miR-145 and thus alter ADAMTS5, while also inhibiting SP1 transcription factor activity and regulating nuclear factor kappa-light-chain-enhancer of activated B cells (NF-κB) in chondrocytes. These factors are orchestrated to promote inflammation and apoptosis but inhibit proliferation in a fine-tuned manner (15). Notably, the targets of miR-145 (SP1 and NF-κB) and miR-146a (PI3K/Akt/mTOR) interact to form an almost complete clique (**Figure 1B**), suggesting a connection between the molecular functions regulated by lncRNA MALAT1 that would not have been identified without integrating the curated data. Aside from MALAT1, lncRNAs SNHG15 (16, 17), PVT1 (18, 19), XIST (20, 21), PART1 (22, 23), and NEAT1 (24, 25) are each shown to have two miRNA targets, which in turn regulate multiple genes with annotated protein-protein interactions (**Figure 1A**). This network illustrates non-coding RNA function in human OA chondrocytes, the complexities of which are not readily apparent when interpreting studies separately.

## Non-coding RNA Network in RA FLS

**Figure 2:**
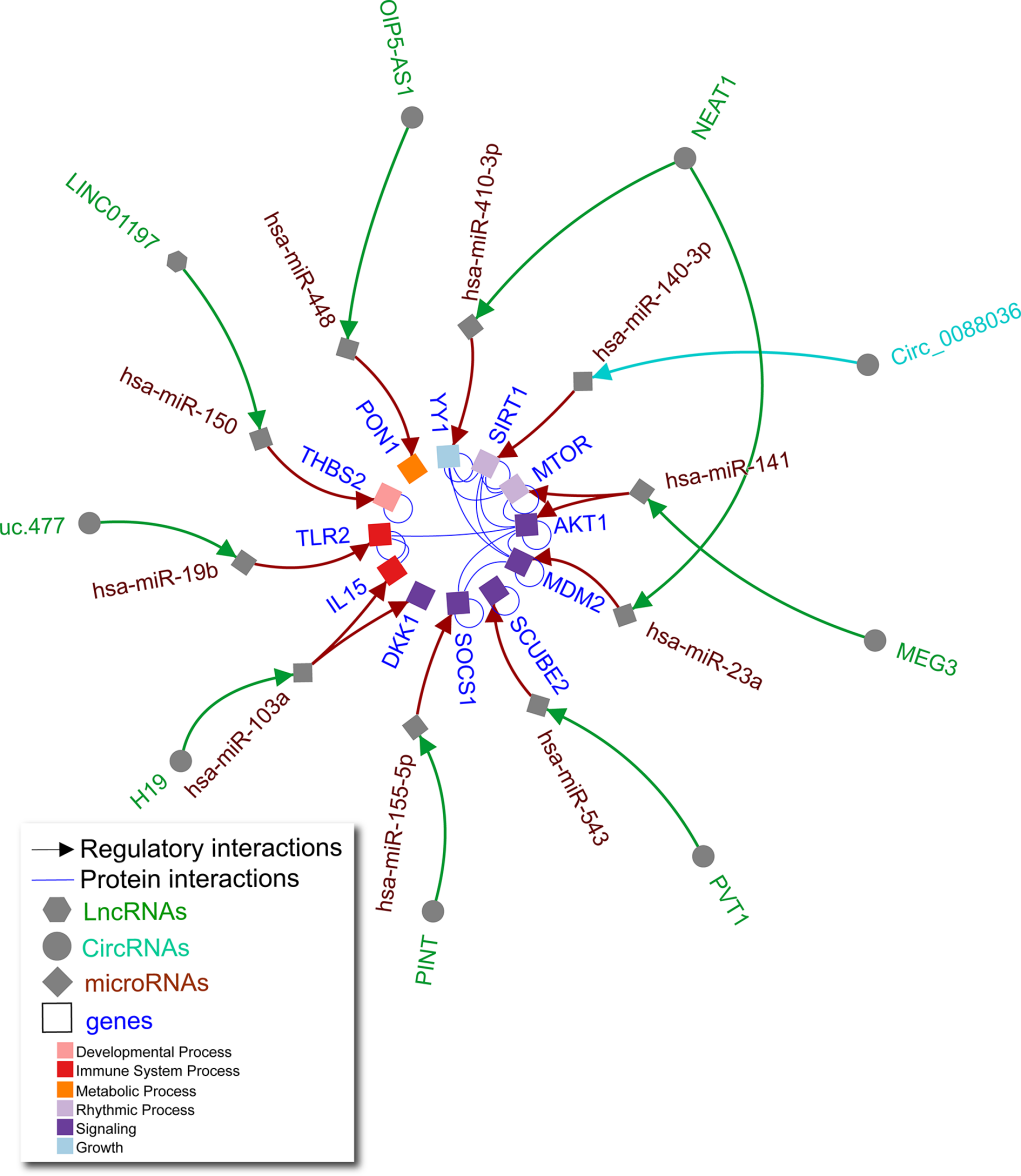
Non-coding RNA network in RA FLS. Literature curated lncRNA-miRNA-gene and circRNA-miRNA-gene axes (green, turquoise, and brown arrows) complemented by physical protein interactions (blue nondirectional lines connecting gene products) showing an interconnected regulatory network of non-coding RNAs in human RA FLS. Node shape represents different molecule types, while gene target color signifies Gene Ontology biological process (as indicated in the legend).

RA is a chronic systemic autoimmune disease that causes inflammation in synovial joints. It affects 0.5% of the global population (26). Like OA, RA can cause cartilage and bone damage, but the major pathological changes in RA are within the synovium. This includes infiltration of immune cells and wildly proliferating invasive FLS exhibiting impaired apoptosis and proinflammatory cytokine production (27). Noncoding RNAs are known to play important roles in RA FLS. Early studies found a dysregulation of miR-155 and miR-146a influenced the proliferative, invasive, and proinflammatory phenotype of RA FLS (28–31). Recent studies suggest a more complex picture of non-coding RNAs within RA FLS (**Figure 2** and **Supplementary Table 2**).

Interactions between lncRNAs, circRNAs, and miRNAs contribute to FLS abnormalities typical of RA (**Figure 2**). For example, lncRNA NEAT1 affects several miRNAs as a significant driver of RA joint pathology. NEAT1 is increased in RA versus control subject synovial tissue (32) and peripheral blood mononuclear cell exosomes (33). These exosomes can deliver additional NEAT1 to FLS (33). NEAT1 sponges miR-410-3p leading to increased YY1 transcription factor which promotes FLS proliferation, impaired apoptosis, and increased tumour necrosis factor alpha (TNF-α) and matrix metallopeptidase 9 (MMP-9) expression (32). NEAT1 also causes increased FLS proliferation and inflammation through sponging miR-23a, leading to an increase in murine double minute-2 (MDM2) (33). This demonstrates that lncRNA NEAT1 can function through different miRNAs to alter expression of unique targets that are further connected by protein-protein interactions as shown for YY1 and MDM2 in **Figure 2**.

Other key lncRNA axes have been reported in RA. For example, excess lncRNA H19 in FLS sponges miR-103a, leading to increased expression of multiple direct targets including IL-15 and dickkopf WNT signaling pathway inhibitor 1 (DKK1), promoting inflammation and joint destruction in RA (34). Downregulated lncRNAs LINC-PINT, LINC01197, and OIP5-AS1 in RA synovial tissue lead to increased levels of their respective targets miR-155, miR-150, and miR-448 in RA FLS, which in turn lead to cell proliferation, invasion, and inflammatory responses (35–37). Upregulated lncRNAs PVT1 and uc.477 lead to sponging of miR-543 (38) and miR-19b (39), respectively, also contributing to RA FLS pathology. While fewer circRNA axes have been elucidated, one study found excess circ0088036 could sponge miR-140-3p to promote proliferation and migration in FLS *via* sirtuin 1 (SIRT1) (40). As in OA chondrocytes, these studies demonstrate the importance of interconnectivity of non-coding RNAs and their gene targets in human RA FLS.

## Discussion

We hold the perspective that non-coding RNAs function in tissue-specific networks that can be elucidated using curation and computational biology to integrate experimentally validated data from individual studies. Taking OA chondrocytes and RA FLS as two examples, we integrated non-coding RNA axes by connecting common molecules and produced networks highlighting connections that may have otherwise been unknown. In particular, miRNAs are shown to orchestrate the effects of lncRNAs and circRNAs on gene expression, which has implications for downstream signaling, including protein-protein interactions and regulation of biological processes. While we did not explore upstream factors regulating the expression of lncRNAs and circRNAs, nor the direct effects of lncRNAs and circRNAs on gene expression, these are equally important considerations for understanding the fine-tuning that non-coding RNAs drive in disease. Since non-coding RNAs are highly stable in circulation, they can be used as diagnostic biomarkers for patients with OA, RA, and other musculoskeletal diseases (41–43). Additionally, we demonstrate that as ceRNAs, lncRNAs and circRNAs can regulate one or more miRNAs, suggesting that miRNAs are important coordinating signals and therefore potential therapeutic targets (44). Targeting non-coding RNAs with antisense oligonucleotides (ASOs) and small interfering RNAs (siRNAs) to selectively modulate their expression in specific tissue types could have therapeutic benefit for OA and RA (45).

To note limitations, our restricted literature search may have missed other reports on the molecules included in the networks, and therefore these are not exhaustive networks. Based on the high number of studies reporting non-coding RNA axes in OA chondrocytes and RA FLS, we focused on these cell types and excluded articles reporting non-coding RNA axes in other cells or biofluids for these diseases. Similarly, to maximize physiological relevance, we chose to exclude studies that used only animal models or cell lines, instead focusing on studies that used primary human chondrocytes or FLS (often in addition to cell lines). By applying these criteria, our intention was to capture axes that were established in comparable biological contexts in order to justify integration across studies. While experimental validation of the networks shown in **Figures 1** and **2** is required, we have pruned the specific species, cell (chondrocytes and FLS, respectively), and disease (OA and RA, respectively) contexts in which these studies should be conducted. This represents a possible workflow in which computational biology methods can inform experimental approaches in non-coding RNA research.

In constructing these non-coding RNA networks, we encountered key challenges pertaining to naming conventions, database maintenance, and target predictions. Assigning names to new molecules has historically led to non-standard nomenclature. The most appropriate approach to limit inconsistent naming is to create standard rules, as evidenced by improved gene naming after the HUGO Gene Nomenclature Committee (HGNC) released guidelines (https://www.genenames.org/about/guidelines/) (46). Unfortunately, non-coding RNAs suffer from naming conventions that are either out-of-date or absent altogether (47, 48). The miRBase reference database has been used for miRNAs since 2004 (49), and this led to fairly accurate miRNA nomenclature in the subsequent 10 years. However, miRbase releases are becoming sparser (the latest being in 2018) and include a small percentage of all the novel miRNAs identified in the literature using sequencing techniques, leading to novel miRNAs being named inconsistently. While irregular and infrequent database updates create a challenge (50), miRNA nomenclature is still better defined than that of other non-coding RNAs.

HGNC provides naming rules for lncRNAs (derived from lncRNAdb, which is no longer active), but not for circRNAs, though some suggestions are provided (48). LNCipedia (https://lncipedia.org) provides consistent nomenclature and links to alternative names, a feature that is useful for mapping different publications to the same molecule (51). Unfortunately, the most recent update was in 2018. CIRCpedia (https://www.picb.ac.cn/rnomics/circpedia/), a reference database for circRNAs, had a similar fate, with no up-to-date curations (52). This lack of nomenclature standards for lncRNAs and circRNAs leads to considerable variability in naming as shown in our networks, and represents a major roadblock to comprehensive data integration from the literature and across databases (53). RNAcentral (https://rnacentral.org/) is a database that is attempting to overcome such roadblocks, integrating data from multiple reference databases (54). However, many of the databases lack updates, and the type of multiple mapping provided for the molecules is not conducive to high-throughput workflows or computational analyses.

With respect to target predictions, miRNA-target predictions have been the focus of several tools and databases over the past 20 years. Among the pitfalls, many of these databases are not being updated, some for more than 10 years (55). This leads to a lack of interactions for more recent miRNAs, including both novel miRNAs and miRNAs in miRbase updates, creating a bias for computational analyses. For lncRNAs and circRNAs, very few databases curate or predict interactions, and of the few that were created in the past decade, many are already no longer available. The largest lncRNA database is LncRNA2Target (http://123.59.132.21/lncrna2target/index.jsp), with 2,189 lncRNA-target interactions derived from low-throughput experiments and 203,500 from high-throughput experiments, and with a recent update in 2021 (56). CircATLAS (http://bio-annotation.cn/lncrna2target/) has annotated 421,501 predicted human circRNAs and provides predicted RNA-binding proteins and miRNA interactions with circRNAs (57). While circATLAS is well organized and provides tissue annotation for circRNAs, one limitation is that it lacks literature curated data.

Although data curation is a lengthy process that requires human expertise and dedicated funds, it is necessary to enable trustable and useful computational biology predictions. A pre-selection of the candidate papers to be curated is necessary to reduce the burden of manual curation. To do this, some databases [e.g., IMEx and GrainGenes (58)] prioritize papers from topic-specific journals. Many databases allow researchers to submit papers to be curated, or to curate the data themselves; however, that may result in inconsistencies or varying level of detail for included information. Multiple efforts using artificial intelligence and text mining have been used over the years to aid the curation process to either pre-filter information to be curated, or automate its annotation [e.g., Wormbase (59)]. However, as already highlighted, naming and other inconsistencies create challenges that must be handled manually.

Since database infrastructure and curation funding are becoming more difficult to obtain, this in part explains the lack of database maintenance and availability, and even data source availability. One approach to promote accurate and up-to-date curation is to create consortia among the groups behind the many databases [as proven by the International Molecular Exchange (IMEx) Consortium for protein interactions, for example (60, 61)]. The scientific community could contribute to this effort by following naming rules and providing links to databases when describing already curated molecules; in turn, databases should agree on one nomenclature and not create multiple unique ones, and should provide mapping for molecules already described using several different identifiers. Again, the protein interaction field provides a successful example of this (62–64). As non-coding RNA research continues to grow, this strategy is expected to support data integration across studies, and in turn increase the value of biological data for translational research by improving reproducibility and interpretability.

While overcoming data integration challenges is not trivial, the networks achieved can provide more complete and more precise insight into the molecular background of a disease of interest. We demonstrate this using an integrative computational biology approach, constructing lncRNA/circRNA-miRNA-mRNA networks in OA chondrocytes and RA FLS. Given the recent surge in studies exploring non-coding RNA axes in various diseases, placing findings of individual studies into a broader biological context while maintaining key parameters (e.g., cell types) is expected to advance our understanding of the net effect of manipulating a single non-coding RNA. From larger networks, sub-networks may be selected for future investigation based on a specific hypothesis, or simply based on the number or nature of connections. Moving forward, concerted efforts to unify nomenclature, maintain databases, and improve context-specific target predictions is expected to support integrative approaches that expedite prioritization of the most promising candidates for experimental exploration in specific biological contexts.

## Data Availability Statement

Data available upon request to the authors as described in methods section.

## Author Contributions

SA and IJ conceptualized the article. SA wrote the first draft with input from NK. CP contributed content on data integration. MP performed the literature search and contributed content on osteoarthritis. MO performed the literature search and contributed content on rheumatoid arthritis. IJ prepared the network figures. All authors critically revised the article and approved the final draft.

## Funding

MP was supported through a Wellcome Trust Clinical Intermediate Fellowship (grant 107471/Z/15/Z). MJO was supported by VA Clinical Science Research and Development award (CDA #IK2 CX001269). IJ was supported in part by funding from Natural Sciences Research Council (NSERC #203475), Canada Foundation for Innovation (CFI #225404, #30865), Ontario Research Fund (RDI #34876), IBM, Ian Lawson van Toch Fund, and the Schroeder Arthritis Institute *via* the Toronto General and Western Hospital Foundation, University Health Network. The funders had no role in study design, data collection and analysis, decision to publish, or preparation of the manuscript.

## Conflict of Interest

SA declares a filed US Provisional Patent Application No. 63/033,463 titled “Circulating MicroRNAs in Knee Osteoarthritis and Uses Thereof”.

The remaining authors declare that the research was conducted in the absence of any commercial or financial relationships that could be construed as a potential conflict of interest.

## Publisher’s Note

All claims expressed in this article are solely those of the authors and do not necessarily represent those of their affiliated organizations, or those of the publisher, the editors and the reviewers. Any product that may be evaluated in this article, or claim that may be made by its manufacturer, is not guaranteed or endorsed by the publisher.
